# Prediction of grading of ovarian endometrioid carcinoma using conventional MRI features

**DOI:** 10.1007/s11604-024-01727-9

**Published:** 2024-12-28

**Authors:** Masaya Kawaguchi, Hiroki Kato, Tatsuro Furui, Masanori Isobe, Yoshifumi Noda, Fuminori Hyodo, Tatsuhiko Miyazaki, Masayuki Matsuo

**Affiliations:** 1 Department of Radiology, Gifu University, 1-1 Yanagido, Gifu, 501-1194 Japan; 2 Department of Radiology, Ogaki Municipal Hospital, 4-86 Minaminokawacho, Ogaki, 503-0864 Japan; 3 Department of Obstetrics and Gynecology, Gifu University, Gifu, Japan; 4 Enter for One Medicine Innovative Translational Research (COMIT), Institute for Advanced Study, Gifu University, Gifu, Japan; 5 Department of Pathology, Gifu University, Gifu, Japan

**Keywords:** Carcinoma, Endometrioid, MRI, Ovary, Tumor grading

## Abstract

**Objective:**

The purpose of this study was to evaluate MRI findings of ovarian endometrioid carcinoma (OEC) as a predictor of histological grade.

**Materials and methods:**

This study included 60 patients with histopathologically confirmed OEC (20, 30, and 10 with grades 1, 2, and 3, respectively). Clinical and MRI results were retrospectively reviewed. We compared the following parameters between the three grades: age, tumor markers, presence of uterine corpus cancer, bilaterality, configuration, peritoneal dissemination, abnormal ascites, signal intensities of cystic and solid components, tumor size, and apparent diffusion coefficient (ADC) values of solid components.

**Results:**

T1-hyperintense cysts were more common in grade 1 than in grades 2–3 OEC (80% vs. 60%, vs. 40%, *p* < 0.05). The signal intensity ratio between the cystic components with the largest solid component and muscle (1.49 vs. 1.08 vs. 0.98, *p* < 0.05) was higher in grade 1 than in grades 2–3 OEC. Necrosis within solid components was less common in grade 1 than in grades 2–3 OEC (31% vs. 68% vs. 88%, *p* < 0.05), and the ADC values of solid components were higher in grade 1 than in grades 2–3 OEC (1.10 vs. 0.99 vs. 0.79 × 10^−3^ mm2/sec,* p* < 0.05). There were no significant differences in other factors.

**Conclusion:**

On T1-weighted images, grade 1 OEC showed a higher signal intensity in the cystic components than grades 2–3 OEC. Necrosis and lower ADC values were more frequently observed in grades 2–3 than in grade 1 OEC.

## Introduction

Ovarian endometrioid carcinoma (OEC) is a malignant epithelial tumor that histologically resembles endometrioid carcinoma of the uterus. It accounts for approximately 10% of all ovarian carcinomas, making it the second most common subtype of epithelial ovarian cancer following high-grade serous carcinoma [1, 2]. OEC primarily affects women with an average 55 year, with endometriosis identified as a significant risk factor [1].

OEC is graded based on the percentage of non-squamous solid components: grade 1 (≤ 5%), grade 2 (6–50%), and grade 3 (> 50%). Nuclear atypia excessive for the grade raises the grade of grade 1 or 2 by one [3, 4]. Grades 2 and 3 OECs share a similar prognosis, immunophenotype, or clinical parameters, while grade 1 exhibits better outcomes or different immunophenotype [3]. Thus, the previous study suggested that grade 2 and 3 OECs could be combined into a single group [3]. The National Comprehensive Cancer Network Clinical Practice Guidelines recommend observation for patients with surgically staged IA–IB, grade 1 tumors. For stage IA–IB, grades 2–3 and stage IC, grade 1 tumors, either observation or platinum-based chemotherapy is advised, while systemic adjuvant chemotherapy is recommended for stages IC–IV tumors [2, 5, 6]. In addition, a recent study showed that staging lymphadenectomy in grade 2 OEC patients was associated with improved disease-free survival and overall survival; however, that in grade 1 OEC was not associated [7]. Therefore, appropriate grade categorization for differentiating between grade 1 and grades 2–3 OECs is essential in terms of selecting proper treatment strategies or predicting prognosis.

MRI typically reveals OEC as an endometriotic cyst with mural nodules, with solid components appearing multicentric and centripetal [8–10]. Although prior studies have compared MRI features of OEC to those of ovarian clear cell or serous carcinoma [11–16], no studies to date have specifically examined the MRI differences between the three grades. This study aims to address this gap by comparing MRI characteristics across these OEC grades.

## Methods

### Patients

This retrospective study, approved by the institutional review board and compliant with the Health Insurance Portability and Accountability Act of 1996, analyzed cases from April 2008 to February 2024. We searched our two Japanese hospital’s electronic medical record system for patients, and inclusion criteria were as follows: (1) surgically resected cases, (2) histologically diagnosed cases, and (3) patients who underwent preoperative MRI. The exclusion criteria were as follows: (1) histologically diagnosed ovarian metastasis from uterine endometrial cancer and (2) inappropriate image quality for evaluation. In total, 60 patients with histopathologically confirmed OEC were included. Patients ranged in age from 22 to 94 years, with a median age of 52. Of these, 20, 30, and 10 patients had grades 1, 2, and 3 OEC, respectively.

### MRI protocols

MRI was conducted using a 1.5-T (Intera Achieva 1.5-T Pulsar; Philips Healthcare, Best, The Netherlands), a 1.5-T (Inginea prodiva 1.5-T CS; Philips Healthcare, Best, The Netherlands), a 1.5-T (GENESIS SIGNA; GE Healthcare, The United States), a 3.0-T (Intera Achieva 3.0 T Quasar Dual; Philips Healthcare, Best, The Netherlands), or 3.0-T scanners (Inginea 3.0 T CX; Philips Healthcare, Best, The Netherlands). Forty-nine patients underwent unenhanced and contrast-enhanced MRI, while 11 patients had only unenhanced imaging. Imaging parameters included a section thickness of 4–7 mm with 1–2 mm intersection gap and a 24 × 24–44 × 44-cm field of view. Acquisitions included axial and sagittal T2-weighted fast spin-echo (TR/TE, 2,700–6423/81–130 ms), axial T1-weighted spin-echo (TR/TE, 367–808/7–11 ms), and axial fat-suppressed T1-weighted fast spin-echo (TR/TE, 367–808/7–10 ms) sequences. In addition, axial diffusion-weighted single shot spin-echo echo-planar (TR/TE, 2,700–5,000/69–80 ms; *b*-value = 0 and 1000 s/mm^2^) was performed in 52 patients, axial and coronal or sagittal fat-suppressed gadolinium-enhanced T1-weighted spin-echo (TR/TE, 367–816/6.7–12 ms) imaging was obtained in 49 patients following the intravenous injection of 0.1 mmol/kg gadopentetate dimeglumine (Magnevist, Bayer HealthCare, Leverkusen, Germany) or gadobutrol (Gadavist, Bayer HealthCare, Leverkusen, Germany).

### Imaging analysis

Two radiologists, with 25- and 11-year of experience in gynecological imaging, independently evaluated all MRI scans. Discrepancies were resolved by consensus. Reviewers were blinded to clinical and pathological data.

First, the reviewers conducted a qualitative assessment of each case by evaluating several parameters, including the margin (well-defined or ill-defined), configuration (pure solid, mixed solid and cystic, or pure cystic), and predominance (cystic or solid). If the EOCs involved bilateral ovaries, the reviewer evaluated the imaging findings of larger lesion. In addition, they assessed the presence of uterine corpus cancer, uterine adenomyosis, pelvic endometriosis, lymphadenopathy, abnormal ascites, and peritoneal dissemination. Lymphadenopathy was defined as a short-axis diameter > 8 mm within the pelvis. Peritoneal dissemination was identified by nodular or smooth thickening of the peritoneum. Abnormal ascites was defined as ascites exceeding the uterine fundus and/or filling the pelvic cavity [17, 18].

Second, the reviewers evaluated the qualitative imaging findings of the cystic component. Parameters included loculation (unilocular or multilocular), signal intensity on T1- (low, iso-, or high) and T2- (iso-, mildly high or high) weighted images, T1-hyperintense cyst, and fluid–fluid levels were evaluated. The signal intensity of the cystic component was evaluated at the cyst with the largest solid component and was compared with that of the iliopsoas muscle. If the lesion had multiple cysts within the tumors, T1-hyperintense cyst was positive when at least one hyperintense cyst relative to the iliopsoas muscle was observed on T1-weighted images.

Third, the qualitative imaging findings for solid components were evaluated based on exophytic growth, mural nodule growth pattern (eccentric or centripetal), surface lobulation, signal intensity (iso-, mildly high, or high), and homogeneity (homogeneous or heterogeneous) on T1- and T2-weighted images. Eccentric and centripetal pattern were defined as numerous solid components existed along the inner cystic surface and a few solid proportions in the cystic mass, respectively [8]. Reviewers noted T2-hyperintense and T2-hypointense areas within the solid components. Signal intensity of solid components was compared with the iliopsoas muscle on T1- and T2-weighted images. If multiple solid components were present, the largest solid component was analyzed. T2-hyperintense or T2-hypointense solid component was present if any hyperintense or hypointense area was observed within the solid component on T2-weighted images. On contrast-enhanced T1-weighted images, enhancement degree (marked or mild), homogeneity (homogeneous or heterogeneous), and necrosis were recorded. Necrosis was defined as ill-defined, unenhanced regions within solid components.

Finally, the quantitative imaging findings included the maximum diameter of the whole tumor and solid component, the height of the solid component, the signal intensity ratio of cystic and solid components on T1- and T2-weighted images, and the signal intensity ratio of solid component on contrast-enhanced T1-weighted images. The apparent diffusion coefficient (ADC) value of the solid component was also measured. Regions of interest (ROIs) were placed on both the cystic and solid components as well as the iliopsoas muscle to calculate signal intensity ratios on T1-, T2-, and contrast-enhanced T1-weighted images. Solid components on contrast-enhanced T1-weighted images were considered as enhanced areas by referring to unenhanced T1-weighted images. The ratio of cystic or solid components to the muscle signal intensity was calculated. Signal intensity of cystic and solid component was measured by placing ROIs on the cyst with the largest solid component and the largest solid component, respectively. The ADC value was determined using ADC maps by placing ROIs within the largest solid component, excluding necrotic and cystic areas based on T2- and contrast-enhanced T1-weighted images [18].

### Statistical analysis

All statistical analyses were performed using EZR (Saitama Medical Center, Jichi Medical University, Saitama, Japan) [19]. Quantitative results of grade 1 and grade 2–3 OECs were compared using the Mann–Whitney *U* test and those of three grades were compared using the Kruskal–Wallis test, while qualitative results were analyzed using Fisher’s exact test. Receiver operation characteristics (ROC) curve analysis was used to determine the performance of quantitative imaging findings, and area under the curve (AUC) was calculated to establish the optimal cutoff value for differentiating grade 1 OEC from grade 2–3 OECs. The optimal ROC cutoff value was determined based on the Youden criterion. Moreover, the sensitivity, specificity, and accuracy for differentiating grade 1 OEC from grade 2–3 OECs were calculated. A *p* value < 0.05 was considered statistically significant. Inter-observer variability for qualitative assessments was measured using kappa statistics.

## Results

The clinical findings for OECs are summarized in Table 1. There were no significant differences between the three histological grades with respect to age, tumor markers, staging, and coexisting conditions.

**Table 1:** The clinical findings of OEC

	All OECs (*n* = 60)	G1 (*n* = 20)	G2 (*n* = 30)	G3 (*n* = 10)	*p* value	
					G1 vs. G2 vs. G3	G1 vs. G2–3
Age (year)	52 [48–63]	50 [46–61]	52 [48–64]	54 [51–62]	0.76	0.65
Tumor marker						
CA 19–9 (U/mL)	52 [19–530]	95 [27–1803]	48 [10–592]	21 [10–69]	0.18	0.12
CA 72–4 (U/mL)	2.7 [10–51]	11 [4.0–29]	11 [3.1–56]	8.3 [5.1–42]	0.97	0.85
CA-125 (U/mL)	139 [62–840]	114 [41–841]	163 [52–868]	233 [125–645]	0.64	0.49
FIGO stage						
I	37 (62)	15 (75)	19 (63)	3 (30)	0.10	0.56
II	7 (12)	1 (5)	3 (10)	3 (30)		
III	11 (18)	3 (15)	4 (13)	4 (40)		
IV	5 (8)	1 (5)	4 (13)	0 (0)		
Coexisting conditions						
Uterine corpus cancer	26 (43)	9 (45)	12 (40)	5 (50)	0.83	> 0.99
Endometriosis	21 (35)	6 (30)	14 (47)	1 (10)	0.11	0.78

Quantitative data are expressed as medians with interquartile in square brackets

Qualitative data are expressed as raw numbers with percentages in parentheses

*OEC* ovarian endometrioid carcinoma, *FIGO* The International Federation of Gynecology and Obstetrics

The imaging findings of OEC are summarized in Table 2. There were no significant differences in bilaterality, margin, configuration, predominance, uterine corpus cancer, uterine adenomyosis, pelvic endometriosis, lymphadenopathy, abnormal ascites, or peritoneal dissemination between the three histological grades.

**Table 2:** The imaging findings of OEC

	All OECs (*n* = 60)	G1 (*n* = 20)	G2 (*n* = 30)	G3 (*n* = 10)	*p* value		Kappa
					G1 vs. G2 vs. G3	G1 vs. G2–3	
Bilaterality	44 (73)	12 (60)	23 (77)	9 (90)	0.22	0.13	
Margin—well defined	56 (93)	19 (95)	28 (93)	9 (90)	> 0.99	> 0.99	0.40
Configuration					0.50	0.33	0.49
Pure solid	1 (2)	1 (5)	0 (0)	0 (0)			
Mixed solid and cystic	59 (98)	19 (95)	30 (100)	10 (100)			
Pure cystic	0 (0)	0 (0)	0 (0)	0 (0)			
Predominance					0.32	0.23	0.66
Cystic	43 (72)	12 (60)	24 (80)	7 (70)			
Solid	17 (28)	8 (40)	6 (20)	3 (30)			
Uterine corpus cancer	23 (38)	7 (35)	10 (33)	6 (60)	0.33	0.78	0.74
Uterine adenomyosis	4 (7)	1 (5)	2 (7)	1 (10)	> 0.99	> 0.99	1.00
Pelvic endometriosis	6 (10)	1 (5)	4 (13)	1 (10)	0.85	0.65	0.84
Lymphadenopathy	3 (5)	1 (5)	2 (7)	0 (0)	> 0.99	> 0.99	0.49
Abnormal ascites	15 (25)	4 (20)	8 (27)	3 (30)	0.79	0.75	0.51
Peritoneal dissemination	9 (15)	1 (5)	6 (20)	2 (20)	0.27	0.25	0.56

Qualitative data are expressed as raw numbers with percentages in parentheses

*OEC* ovarian endometrioid carcinoma

**Fig. 1:**
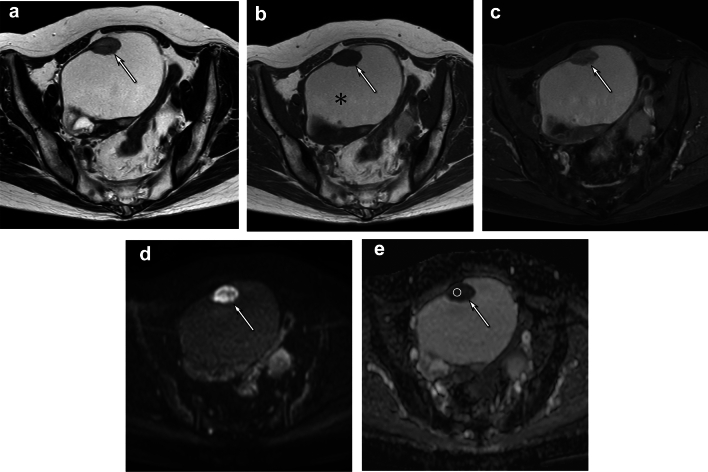
A 66-year-old female with grade 1 OEC. **a** Axial T2-weighted image showing a cystic mass with solid components (arrow). **b** Axial T1-weighted image showing a cystic mass with solid components (arrow). The cystic component displays high signal intensity relative to the muscle (asterisk). **c** Axial fat-suppressed contrast-enhanced T1-weighted image showing homogeneous enhancement of the solid component (arrow). **d** Axial diffusion-weighted image showing solid component with high signal intensity. **e** Axial ADC map indicating restricted diffusion (ADC value; 1.11 × 10^−3^ mm.^2^/sec)

**Fig. 2:**
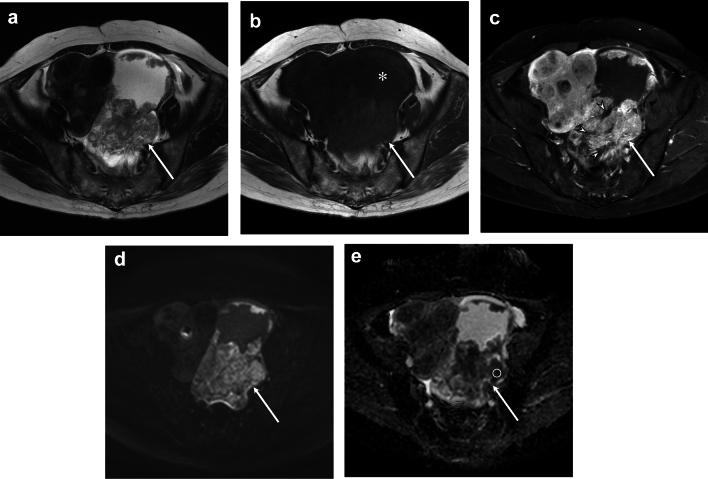
A 52-year-old female with grade 2 OEC. **a** Axial T2-weighted image showing a cystic mass with heterogeneous solid components (arrow). **b** Axial T1-weighted image showing a cystic mass with solid components (arrow). The cystic component displays iso-signal intensity relative to the muscle (asterisk). **c** Axial fat-suppressed contrast-enhanced T1-weighted image showing heterogeneous enhancement of solid component (arrow) with necrosis (arrowhead). **d** Axial diffusion-weighted image showing a solid component with high signal intensity. **e** Axial ADC map indicating restricted diffusion (ADC value; 0.97 × 10^−3^ mm.^2^/sec)

Table 3 presents the imaging findings of cystic components of OECs. T1-hyperintense cysts were prevalent in grade 1 OEC than in grades 2–3 OEC (80% vs. 60% vs. 40%, *p* < 0.05) (Figs. 1, 2). Signal intensity of the cystic component with the largest solid component on T1-weighted images was low in 2, 8, and 3 cases, iso- in 2, 7, and 5 cases, and high in 15, 15, and 2 cases for grade 1, 2, and 3 OEC, respectively (*p* < 0.05). There were no significant differences in loculation, signal intensity on T2-weighted images, or fluid–fluid levels between the three histological grades.

**Table 3:** Imaging findings for the cystic and solid component of OEC

	All OECs (*n* = 60)	G1 (*n* = 20)	G2 (*n* = 30)	G3 (*n* = 10)	*p* value		Kappa
					G1 vs. G2 vs. G3	G1 vs. G2–3	
*Cystic component*	*n* = 59	*n* = 19	*n* = 30	*n* = 10			
Loculation—multilocular	37 (63)	10 (53)	18 (60)	9 (90)	0.13	0.39	0.67
The signal intensity on T1WI^1^					0.030*	0.033**	0.60
Low	13 (22)	2 (11)	8 (27)	3 (30)			
Iso-	14 (24)	2 (11)	7 (23)	5 (50)			
High	32 (54)	15 (78)	15 (30)	2 (20)			
T1-hyperintense cysts	36 (61)	16 (80)	18 (60)	4 (40)	0.041*	0.042**	0.85
The signal intensity on T2WI^1^					> 0.99	0.79	0.68
Iso-	2 (3)	1 (5)	1 (3)	0 (0)			
Mildly high	3 (5)	1 (5)	2 (7)	0 (0)			
High	54 (92)	17 (89)	27 (90)	10 (100)			
Fluid–fluid levels	10 (17)	2 (11)	6 (20)	2 (20)	0.65	0.48	0.52
*Solid component*	*n* = 60	*n* = 20	*n* = 30	*n* = 10			
Exophytic growth	8 (13)	2 (10)	5 (17)	1 (10)	0.88	0.71	0.48
Mural nodule growth pattern					0.33	0.35	0.35
Eccentric	14 (23)	3 (15)	7 (23)	4 (40)			
Centripetal	46 (77)	17 (85)	23 (77)	6 (60)			
Lobulated surface	47 (78)	14 (70)	23 (77)	10 (100)	0.14	0.33	0.51
The signal intensity on T1WI					0.72	0.45	0.50
Iso-	47 (78)	16 (80)	23 (77)	8 (80)			
Mildly high	12 (20)	3 (15)	7 (23)	2 (20)			
High	1 (2)	1 (5)	0 (0)	0 (0)			
Homogeneous on T1WI	39 (65)	12 (60)	19 (63)	8 (80)	0.67	0.58	0.39
The signal intensity on T2WI					0.83	0.70	0.85
Iso-	1 (2)	0 (0)	1 (3)	0 (0)			
Mildly high	57 (95)	20 (100)	27 (90)	10 (100)			
High	2 (3)	0 (0)	2 (7)	0 (0)			
T2-hyperintensity	30 (50)	8 (40)	16 (53)	6 (60)	0.58	0.41	0.47
T2-hypointensity	6 (10)	1 (5)	4 (13)	1 (10)	0.85	0.65	0.50
Homogeneous on T2WI	8 (13)	2 (10)	4 (13)	2 (20)	0.77	0.71	0.20
*Contrast-enhanced T1WI*	*n* = 49	*n* = 13	*n* = 28	*n* = 8			
Enhancement degree—marked	7 (14)	1 (8)	5 (18)	1 (13)	0.85	0.66	0.50
Homogeneity–Homogeneous	9 (18)	4 (31)	4 (14)	1 (13)	0.47	0.22	0.36
Necrosis	30 (61)	4 (31)	19 (68)	7 (88)	0.023*	0.018**	0.46

Quantitative data are expressed as medians with interquartile in square brackets. Qualitative data are expressed as raw numbers with percentages in parentheses

*OEC* ovarian endometrioid carcinoma

^1^Signal intensity of the cystic component was assessed at the cyst with the largest solid component

*Significant difference was observed between the three histological grades (*p* < 0.05). **Significant difference was observed between G1 and G2–3 OECs (*p* < 0.05).

Imaging findings of solid components of OECs are summarized in Table 3. There were no significant differences between the three histological grades in exophytic growth, mural nodule growth pattern, lobulated surface, signal intensity, homogeneity, or T2- hyperintensity/hypointensity. On contrast-enhanced T1-weighted images, necrosis was less frequent in grade 1 than in grades 2–3 OEC (31% vs. 68% vs. 88%, *p* < 0.05) (Figs. 1, 2). There were no significant differences in enhancement degree or homogeneity between the three histological grades.

Quantitative imaging findings are summarized in Table 4. The cystic component with the largest solid component exhibited a higher signal intensity ratio relative to muscle in grade 1 OEC compared with grades 2–3 OEC on T1-weighted images (1.49 vs. 1.08 vs. 0.98, *p* < 0.05). The ADC value of the solid component was significantly higher in grade 1 OEC than in grades 2–3 OEC (1.10 vs. 0.99 vs. 0.79 × 10^−3^mm^2^/sec,* p* < 0.05) (Figs. 1, 2). There were no significant differences in the maximum diameter, height of the solid component, or signal intensity ratio of the largest solid component to the muscle on T1-, T2, contrast-enhanced T1-weighted images between the three histological grades.

**Table 4:** Quantitative imaging findings

	All (*n* = 60)	G1 (*n* = 20)	G2 (*n* = 30)	G3 (*n* = 10)	*p* value	
					G1 vs. G2 vs. G3	G1 vs. G2–3
The maximum diameter of the whole tumor (mm)	97 [78–132]	88 [82–148]	102 [78–127]	89 [62–132]	0.79	0.73
The maximum diameter of the solid component (mm)	49 [35–83]	51 [22–86]	48 [41–79]	48 [32–78]	0.68	0.62
The height of the solid component (mm)	33 [20–46]	20 [15–42]	34 [28–50]	34 [22–39]	0.25	0.16
The cystic component^1^	*n* = 59	*n* = 19	*n* = 30	*n* = 10		
SIR on T1WI	1.12 [0.97–1.60]	1.49 [1.19–1.94]	1.08 [0.92–1.49]	0.98 [0.85–1.03]	0.004*	0.006**
SIR on T2WI	5.31 [3.68–6.86]	4.73 [2.81–6.62]	5.34 [3.77–6.89]	5.92 [3.77–6.87]	0.56	0.32
The largest solid component						
SIR on T1WI	1.07 [0.95–1.17]	1.05 [0.94–1.20]	1.08 [0.92–1.18]	1.08 [1.04–1.15]	0.97	0.98
SIR on T2WI	2.99 [2.42–3.74]	3.21 [2.43–3.52]	2.97 [2.23–4.21]	2.94 [2.69–3.77]	0.84	0.90
SIR CET1WI	1.59 [1.50–1.82] (*n* = 49)	1.79 [1.36–1.95] (*n* = 13)	1.57 [1.44–1.76] (*n* = 28)	1.75 [1.61–1.87] (*n* = 8)	0.76	0.73
ADC value (10^−3^mm^2^/sec)	1.02 [0.90–1.13] (*n* = 52)	1.10 [0.95–1.17] (*n* = 18)	0.99 [0.90–1.12] (*n* = 27)	0.79 [0.75–0.97] (*n* = 7)	0.033*	0.024**

Quantitative data are expressed as medians with interquartile in square brackets

Qualitative data are expressed as raw numbers with percentages in parentheses

*OEC* ovarian endometrioid carcinoma, *MD* maximum diameter, *SIR* signal intensity ratio, *ADC* apparent diffusion coefficient

^1^The SIR of the cystic component was assessed at the cyst with the largest solid component

^*^Significant difference was observed between the three histological grades (*p* < 0.05). **Significant difference was observed between G1 and G2–3 OECs (*p* < 0.05)

Diagnostic performance of differentiating grade 1 OEC from grades 2–3 OEC are summarized in Table 5. The ROC analysis revealed that signal intensity ratio of the cystic component on T1-weighted images using an optimal cutoff value of 1.17 had the highest AUC (0.72), followed by ADC value of the solid component using an optimal cutoff value of 0.91 × 10^−3^ mm^2^/sec (0.69). The sensitivity, specificity, and accuracy of diagnosing grade 1 OEC were as follows: T1-hyperintence cyst, 80%, 45%, and 57%, respectively; no necrosis, 69%, 72%, and 71%, respectively; signal intensity of the cystic component with the largest solid component on T1-weighted images (optimal cutoff value > 1.17), 78%, 59%, and 66%, respectively; ADC value (optimal cutoff value > 0.91 × 10^−3^ mm^2^/sec), 94%, 38%, and 58%, respectively.

**Table 5:** Diagnostic performance of differentiating grade 1 OEC from grades 2–3 OECs

	AUC	Sensitivity (%)	Specificity (%)	Accuracy (%)
T1-hyperintense cysts	NA	80.0	45.0	56.7
No necrosis	NA	69.2	72.2	71.4
SIR of cyst on T1WI (> 1.17)	0.72	77.8	58.6	66.0
ADC value (> 0.91)	0.69	94.4	38.2	57.7

*OEC* ovarian endometrioid carcinoma, *SIR* signal intensity ratio, *ADC* apparent diffusion coefficient, *AUC* area under the curve, *NA* not available

## Discussion

T1-hyperintense cysts were more prevalent in grade 1 than in grades 2–3 OEC and the signal intensity ratio of the cyst with the largest solid component to the iliopsoas muscle on T1-weighted images was higher in grade 1 OEC than in grades 2–3 OEC. Solid component necrosis was less common in grade 1 than in grades 2–3 OEC, and the ADC value of the solid component was higher in grade 1. Grades 2–3 OEC were characterized by lower signal intensity of the cystic components on T1-weighted images, solid component necrosis, and lower ADC values of the solid component.

Several previous studies have reported the MRI findings of OEC. OEC typically presents with predominantly cystic lesions, a round or oval shape, hyperintense cystic components on T1-weighted images, and multifocal or centripetal mural nodules [11, 13–15] [20]. The findings of this study, which included the largest population to date for evaluating MRI findings in OEC, are consistent with prior research.

In this study, T1-hyperintense cysts were frequently observed in grade 1 OEC, and the signal intensity ratio of the cystic component with the largest solid component was higher in grade 1 than in grades 2–3 OECs. In other words, signal intensity of the cystic component on T1-weighted images tended to be higher in G1 OEC compared to G2–3 OECs. Previous studies have reported that 35%–85% of the cystic components of OECs exhibits iso-intensity to hyperintensity compared with the muscle [11, 15, 16]. Histopathologically, most OECs originate from endometrial cysts, with polypoid nodules projecting into the lumen of blood-filled cysts [1]. However, no radiological or pathological study has revealed a relationship between tumor grade and the cystic content of OEC. Our results suggest that the hemorrhagic content of the cyst may be less concentrated in grades 2–3 OEC than in grade 1. Moreover, grade 3 OEC and high-grade serous carcinoma share similar pathological morphologies, including glandular or cribriform architecture [3, 21]. As a result, grade 3 OEC may appear as low-signal cyst on T1-weighted images, similar to high-grade serous carcinoma [15, 22]. Although further research is required, the signal intensity of cyst contents on T1-weighted images may aid in grading OEC.

Necrosis within the solid components was less frequent in grade 1 than in grades 2–3 OEC in the present study. Macroscopically, OECs typically exhibit both solid and cystic components with hemorrhage or necrosis present in the solid regions [1]. However, no previous radiological or pathological study has reported correlation between tumor grade and necrosis in OEC. A prior study on uterine endometrial cancer found that pathological necrosis was associated with higher histological grade, more aggressive clinical features, and poor prognosis [23]. Similarly, we propose that the presence of intratumoral necrosis correlates with higher-grade OEC, analogous to uterine endometrial cancer.

The present study also found that the ADC value of the solid components was higher in grade 1 than in grades 2–3 OEC. Previous studies on the diffusion-weighted images characteristics of OEC reported ADC values for the solid components ranging from 0.79 to 1.09 × 10^−3^ mm^2^/s [11, 14, 15]. However, no study has yet investigated the ADC values for different OEC grade. Although several studies on uterine endometrial cancer have explored the relationship between ADC values and tumor grade, the results remain inconclusive. While some studies reported a correlation between ADC values and tumor grade [24–26], others found no relationship [27, 28]. Although further research is necessary, our findings suggest that ADC values may serve as a useful metric for grading OEC.

Diagnostic performance of differentiating between grade 1 and grade 2–3 OECs showed low specificity, especially in ADC value. Using an optimal cutoff ADC value of 0.91 × 10^−3^ mm^2^/sec, 19 patients with grade 2 OEC and 2 with grade 3 were false positive. The considerable overlap of ADC values between grade 1 and grade 2 OECs decreased the specificity. However, since high diagnostic performance was not also obtained for the other imaging findings in this study, further investigation for differentiating between grade 1 and grade 2–3 OECs is required.

This study has several limitations. First, the sample size was relatively small. Second, contrast-enhanced MRI and diffusion-weighted images were not performed in 11 and 8 patients, respectively. Third, dynamic contrast-enhanced MRI could not be evaluated because only 16 patients underwent the procedure. Fourth, diffusion-weighted images data were acquired from five different MRI scanners, as the study included OEC cases from two institutions. Finally, several pathologists were involved in determining tumor grades, given that the samples were collected from two institutions.

In conclusion, this study represents the largest evaluation of MRI findings in OEC to date. On T1-weighted images, grade 1 OEC exhibited a higher signal intensity of the cystic components than grades 2–3 OEC. Necrosis within the solid components was more common in grades 2–3 OEC than in grade 1 OEC, and the ADC values of solid components were lower in grades 2–3 OEC. These MRI findings provide essential insights into the differentiation of grade 1 OEC from grades 2–3, contributing to more accurate preoperative diagnosis and improved treatment strategies.

## Declarations

### Data availability

Data will be made available on reasonable request.
